# Synthetic Cationic Peptide IDR-1002 and Human Cathelicidin LL37 Modulate the Cell Innate Response but Differentially Impact PRRSV Replication *in vitro*

**DOI:** 10.3389/fvets.2019.00233

**Published:** 2019-07-12

**Authors:** Benoît Levast, Daniel Hogan, Jill van Kessel, Stacy Strom, Stew Walker, Jianzhong Zhu, François Meurens, Volker Gerdts

**Affiliations:** ^1^Vaccine and Infectious Disease Organization-International Vaccine Centre, University of Saskatchewan, Saskatoon, SK, Canada; ^2^Department of Computer Science, University of Saskatchewan, Saskatoon, SK, Canada; ^3^College of Veterinary Medicine, Comparative Medicine Research Institute, Yangzhou University, Yangzhou, China; ^4^Joint International Research Laboratory of Agriculture and Agri-Product Safety, Yangzhou, China; ^5^BIOEPAR, INRA, Oniris, Nantes, France; ^6^Department of Veterinary Microbiology and Immunology, Western College of Veterinary Medicine, University of Saskatchewan, Saskatoon, SK, Canada

**Keywords:** PRRSV, porcine alveolar macrophages, host defense peptides, poly(I:C), adjuvant

## Abstract

Host defense peptides (HDPs) show both antimicrobial and immunomodulatory properties making them important mediators of the host immune system. In humans but also in pigs many HDPs have been identified and important families such as cathelicidins and defensins have been established. In our study, we assessed: (i) the potential interactions that could occur between three peptides (LL37, PR39, and synthetic innate defense regulator (IDR)-1002) and a common TLR ligand called poly(I:C); (ii) the impact of selected peptides on the response of alveolar macrophage (AM) to poly(I:C) stimulation; (iii) the anti-porcine respiratory and reproductive syndrome virus (PRRSV) properties of the peptides; and (iv) their adjuvant potential in a PRRSV challenge experiment after immunization with different vaccine formulations. The results are as following: LL37, PR39, and IDR-1002 were able to interact with poly(I:C) using an agarose gel migration assay. Then, an alteration of AM's response to poly(I:C) stimulation was observed when the cells were co-stimulated with LL37 and IDR-1002. Regarding the anti-PRRSV potential of the peptides only LL37 showed a PRRSV inhibition in infected AM as well as precision cut lung slices (PCLS). However, in our conditions and despite their immunomodulatory properties, neither LL37 nor IDR-1002 showed any convincing potential as an adjuvant when associated to killed PRRSV in a challenge experiment. In conclusion, both antiviral and immunomodulatory properties could be identified for LL37, only immunomodulatory properties for IDR-1002, and both peptides failed to improve the immune response consecutive to an immunization with a killed vaccine in a PPRSV challenge experiment. However, further studies are needed to fully decipher and explain differences between peptide properties.

## Introduction

Host defense peptides (HDPs) are important effector molecules of the immune system with both antimicrobial and immunomodulatory properties (1–5). Two main families, cathelicidins and defensins, have been described in many species thus far. LL37 and the proline-arginine-rich peptide PR39 are both members of the cathelicidin family with LL37 representing the C-terminal part of the human cationic antimicrobial protein (hCAP) 18 and PR39 being a full-length porcine cathelicidin (2, 4). These two HDPs are expressed by the intestinal and the respiratory mucosa and are produced by epithelial cells, macrophages, and neutrophils (2, 4, 6). Both defensins and cathelicidins have high potential as stand-alone treatments or as vaccine adjuvants (1, 3, 7–9). For instance, LL37 has been clearly described in humans as a regulator of chemokine expression, apoptosis, and responses to a common toll-like receptor (TLR) ligand such as poly(I:C) (9–11). Furthermore, LL37 has been described as antiviral peptides that either interact directly with the viral membrane or stimulate the antiviral immune response against various viruses including adenovirus (12), dengue virus (13), influenza A virus (IAV) (14), herpes simplex virus (HSV) (15), human immunodeficiency virus-1 (HIV-1) (16), respiratory syncytial virus (RSV) (17, 18), and vaccinia virus (19, 20) [for a review see (21)]. Similarly to LL37 in humans, PR39 has been identified as an important effector molecule of the innate immune response in the pig model (4), which has been described as a valuable animal model in vaccine development and testing (22). Like LL37, this HDP is involved in the antimicrobial response, has immunomodulatory properties, and several other biological functions (2, 6, 23).

Because of this wide range of properties, HDPs have been extensively studied and their synthetic innate defense regulator (IDR) peptide analogs have been generated with some of them currently under evaluation for their prophylactic and therapeutic potential (1–3, 24). IDR-1 is a synthetic peptide lacking direct antimicrobial activity but still providing broad-spectrum protection against systemic infections involving for instance multidrug-resistant bacteria. The synthetic peptide IDR-1002(VQRWLIVWRIRK-NH_2_), selected from a library of bactenecin derivatives with a higher potency compared with IDR-1 in inducing chemokines *in vitro*, was previously fully characterized (25). IDR-1002 with IDR-1 and also IDR-1018 are well-known examples of IDRs (24). These synthetic peptides have all an exceptional capacity to stimulate cell recruitment and suppress strong inflammatory responses. Because of these properties IDR-1002 has been further tested and been shown highly effective as an adjuvant when co-formulated with TLR ligands such as poly(I:C), CpG oligodeoxynucleotides, or polyphosphazene (24–26). As the formulations of many new vaccines involve the combination of various adjuvants acting through more than one pathway and stimulating both innate and adaptive immune responses (27, 28), it became crucial to ensure that the components can act synergistically instead of neutralizing each other's activity. In previous studies, conflicting results suggest that HDP can either neutralize TLR ligand agonists (11, 29) or enhance TLR signaling (27, 30, 31).

The porcine respiratory and reproductive syndrome (PRRS) is a major porcine infectious disease worldwide caused by a virus named PRRSV which is a member of the *Arteriviridae* family mostly targeting alveolar macrophages (32). To overcome the infection, the porcine host relies mostly on the adaptive immune response through neutralizing antibodies (NAbs) which appear only late, typically >28 days post-infection (32) and the cellular response, even if it seems to be strain dependent to some extent (33). Thus, usual correlates of protection are the level of serum NAbs measured by seroneutralization assay as well as the number of IFNγ-producing cells measured by ELISPOT (32, 34). To protect against this disease several vaccines have been developed in many countries. However, they have not offered the expected protection thus far and have several limitations including poor protection conferred against heterologous strains of the virus (32). Thus, there is a strong interest in developing new vaccine candidates formulated with optimized adjuvant combinations. Regarding HDPs in the context of PRRSV infection, the literature is quite limited and, to our knowledge, antiviral activity against PRRSV has only been shown for a few HDPs such as protegrin 1 (PG-1), PG-4 and porcine β-defensin 3 (pBD-3) (35, 36).

In that context, the aim of the current study was to briefly assess *in vitro* the immunomodulatory and antiviral properties of the selected peptides (LL37 as a general control showing antiviral properties, PR39 as the full-length porcine cathelicidin, and IDR-1002 as a promising synthetic analog of HDPs) and the adjuvant potential of two most promising peptides in a PRRSV challenge after immunization with different vaccine formulations.

## Materials and Methods

### Virus, Reagents, and Pigs

The virulent PRRSV-2 strain ISU-12-SAH was obtained from ATCC (ATCC VR-2385, Hanassas, VA, USA) and used to carry out the infections. The virus was grown on porcine alveolar macrophages (AM) and the supernatant was stocked at −80°C until use. The virus titer was 10^6^ TCID_50_/mL. Infections were performed at a multiplicity of infection (MOI) of 0.1 for the AMs and with 10^5^ TCID_50_/mL for the precision cut lung slices (PCLS) during 1 h. Then, two washes with phosphate buffered saline (PBS) were carried out. Host defense peptides (HDP) and IDR-1002 were used at a working concentration of 20 μg/mL. Human LL37 was purchased from QCB (Hopkinton, MA, USA), synthetic peptide IDR-1002 was obtained from Dr. Robert Hancock (University of British Columbia, Canada), porcine PR39 was synthetized in house by Dr. Sam Attah-Poku, and poly(I:C) was purchased from Sigma-Aldrich (reference P1530) (St-Louis, MO, USA) and used at 10 μg/mL. All these peptides were regularly tested for the absence of endotoxin contaminations using the LAL Chromogenic Endotoxin Quantitation Kit (Thermo Fisher Scientific, Waltham, MA) per the manufacturer's instruction. For experiments, peptides and virus were mixed 1 h prior to infection of the cells or the tissues. Regarding mock condition, virus was mixed with PBS.

Weaned Dutch Landrace pigs were purchased from Prairie Swine Centre, University of Saskatchewan. The experimental protocol involving the pigs was reviewed and approved by the University of Saskatchewan Animal Care Committee (AUP20030002), which follows the guidelines of the Canadian Council on Animal Care. A total of 44 piglets (5 groups of 8 pigs and 4 pigs to collect tissues and cells) were used in the study. Once the total number of animals and animals per group had been decided a randomization table was generated in an Excel spreadsheet or on-line randomization programs. Numbers were assigned to groups randomly.

### Porcine Alveolar Macrophage Isolation and Culturing

Broncho-alveolar lavages were performed with PBS supplemented with 100 U/mL penicillin and 100 μg/mL gentamycin (GIBCO-BRL, Burlington, ON, Canada). Centrifuged at 300 g and washed two times with PBS, AMs were then cultured in Dulbecco Modified Eagle Medium (DMEM, GIBCO-BRL), supplemented with 100 U/mL penicillin, 100 μg/mL gentamycin and 2% fetal bovine serum (FBS) (GIBCO-BRL). Cell purity was examined by flow cytometry with a staining for CD163 (RPE coupled antibody, AbD Serotec, Raleigh, USA) and SLA class II (FITC coupled antibody, AbD Serotec). The double positive population had up to 90% purity. Cells were incubated overnight and then washed before infection or stimulation.

### Precision-Cut Lung Slices (PCLS)

PCLS were prepared from lungs of 4 eight-week-old pigs. Immediately after euthanasia, lungs were carefully removed and the left cranial, middle, and caudal lobes were filled with 37°C warm low-gelling temperature agarose (Sigma–Aldrich) followed by polymerization on ice. Agarose was dissolved in Roswell Park Memorial Institute (RPMI) 1640 medium (GIBCO-BRL) (1.5% agarose) for lung instillation. Tissue was excised in cylindrical portions (8-mm tissue coring tool) and around 200 slices/pig approximately 250 μm thick were prepared by using a Krumdieck tissue slicer (model MD6000, TSE systems, Chesterfield, MO, USA) with a cycle speed of 60 slices/minute (min). PCLS were incubated in 1 mL of RPMI 1640 medium (GIBCO-BRL), supplemented with 1% antibiotic/antimycotic (Anti-Anti 100x, GIBCO^®^-BRL), clotrimazole 1 μg/mL (Sigma–Aldrich), enrofloxacin 10 μg/mL (Bayer Inc., Toronto, ON), and kanamycin 80 μg/mL (GIBCO-BRL) in a 24-well plate at 37°C and 5% CO_2_. The medium was changed every hour the first four h and once after 24 h, prior to infection. Viability was checked by observing ciliary activity under a light microscope (Olympus CKX31, Tokyo, Japan). In selected samples, slices were analyzed for bronchoconstriction by addition of 10^−4^ M methacholine (acetyl-ß-methylcholine chloride, Sigma-Aldrich), as previously described (37).

### Peptides-Poly(I:C) Complex Formation Test

Interaction of the selected peptides with poly(I:C) was assessed by gel migration delay. Different concentrations of HDP and poly(I:C) were mixed and incubated 30 min at 37°C. Controls and mixed samples were run on a 1% agarose gel and poly(I:C) was detected using ethidium bromide (Sigma–Aldrich). Gels were analyzed using UV light on a Bio-Rad gel imaging detector (ChemiDoc system, Mississauga, ON, Canada).

### Porcine Alveolar Macrophage Stimulation Analysis

Macrophages were stimulated with poly(I:C) at 10 μg/mL with or without the selected peptides. Cells and supernatants were harvested at 6 h (cell pellets—Reverse Transcription-quantitative PCR, RT-qPCR) or 24 h (supernatant—Enzyme-Linked Immunosorbent Assay, ELISA).

For RT-qPCR, mRNA was extracted using a protocol previously described (38). RNA quality was assessed by capillary electrophoresis (Agilent 2100 Bioanalyzer, Santa Clara, CA, USA). Reverse transcription reactions and qPCR assays (for gene targets and primers, see Table 1) were also performed as previously described (38, 39). RNA concentration was determined by measuring optical density (OD) at 260 nm (OD260) and the RNA quality was assessed by calculating OD260/OD280 ratio and by capillary electrophoresis (Agilent 2100 Bioanalyzer, Agilent Technologies Inc., Santa-Clara, USA). cDNA was generated from 100–200 ng of RNA per reaction and RT-PCR was performed using the SuperScript™ III Platinum® Two-Step RT-qPCR Kit as per the manufacturer's recommendations (Invitrogen). The generated cDNA was stored at −80°C. qPCR assays were carried out as previously reported using the two most stable reference genes (38, 40). qPCR data were expressed as relative values after Genex macro analysis (Bio-Rad) (41) using the Cycle quantification (*Cq*) from the samples for the different transcripts.

**Table 1:** List of primers (reference genes are underlined).

**Gene targeted**	**Primer sequences**	**Amplicon sizes (bp)**	**Annealing temperatures**	**Accession numbers**
B2M	*F-TTCTGGTCCACACTGAGTTC*	126	60	*[NM_213978]*
	*R-ATCTCTGTGATGCCGGTTAG*			
CCL2	*F-AGTCACCTGCTGCTATACAC*	117	60	*[NM_214214]*
	*R-GCGATGGTCTTGAAGATCAC*			
CCL3	*F-GCCTGCTGCTTCTCCTATAC*	178	60	*[AY643423]*
	*R-TCAGCTCCAGGTCAGAGATG*			
CCL5	*F-TGCCCTTGCTGTCATCCTC*	201	60	*[NM_001129946]*
	*R-CACACCTGGCGGTTCTTTC*			
IL6	*F-ATCAGGAGACCTGCTTGATG*	177	60	*[NM_214399]*
	*R-TGGTGGCTTTGTCTGGATTC*			
IL8	*F-TCCTGCTTTCTGCAGCTCTC*	100	62	*[NM_213867]*
	*R-GGGTGGAAAGGTGTGGAATG*			
IFNα	*F-CTCCTGGCACAAATGAGGA*	158	60	*[XM_003483387]*
	*R-CTGAAGAGCTGGAAGGTCTG*			
IFNß	*F-GGAACTTGATGGGCAGATGG*	159	60	*[EU744562]*
	*R-CAGGCACAGCTTCTGTACTC*			
RPL19	*F-AACTCCCGTCAGCAGATCC*	147	60	*[AF_435591]*
	*R-AGTACCCTTCCGCTTACCG*			
SOCS1	*F-CGCCCTCAGTGTGAAGATGG*	110	62	*[NM_001204768]*
	*R-GCTCGAAGAGGCAGTCGAAG*			
STAT6	*F-TCCCAGCTACGATCAAGATG*	171	60	*[NM_001197306]*
	*R-AGTGAGAGTGTGGTGGATAC*			
TNFα	*F-CCAATGGCAGAGTGGGTATG*	116	60	*[NM_214022]*
	*R-TGAAGAGGACCTGGGAGTAG*			

For the ELISA (IFNα and TNFα), cells were harvested after 24 h stimulation and centrifuged for 5 min at 400g. IFNα ELISA was performed with a homemade ELISA using an R&D Systems antibody (Minneapolis, MN, USA) as previously described (38). TNFα ELISA was carried out using Porcine TNFα Quantikine ELISA Kit according to the manufacturer's recommendations (R&D Systems).

### Immunization Trial

Dutch Landrace piglets were housed at 3 weeks of age and acclimatized for 1 week before performing the first immunization (day 0) and a boost at day 14. Five groups of pigs (*n* = 8) received either inactivated VR2385-micropraticles (mp) of polyphosphazene (PCEP)-IDR-1002-poly(I:C) using intra-muscular or intra-nasal routes (groups **IDR-1002/IM** and **IDR-1002/IN**) or inactivated VR2385-mpPCEP-LL37-poly(I:C) using intra-muscular or intra-nasal routes (groups **LL37/IM** and **LL37/IN**) or the adjuvant mpPCEP-poly(I:C) alone in IM (group **Controls**). Two weeks after the second immunization (day 28), all pigs were challenged intra-nasally (IN) with PRRSV VR-2385 and body temperature was monitored daily for a total of 7 days until day 6 post-challenge (Supplementary Figure 1). Nasal swabs and blood samples were collected every 3-4 days following challenge. Pigs were euthanized at day 42 and tissues sampled and analyzed. One pig (group LL37/IM) was euthanized before the end of the experiment due to leg injury and inflammation. This pig was not included in the data analysis.

Chemical inactivation was performed on a 1 × 10^5^ TCID_50_/mL PRRSV stock, obtained from infected AM culture supernatant, using 2-bromo-ethylamine intercalate agent (BEI) (42). For one immunization dose, 1x10^5^ TCID_50_ of BEI-treated virus was associated with 10 μg of poly(I:C), 20 μg of IDR-1002 or LL37 peptides, and 10 μg of PCEP (1-2-1 ratio). The adjuvants were complexed by incubating HDP and TLR ligand, then PCEP and virus were assembled as previously described (43–45). Quality of the formulation was evaluated by measuring virus incorporation into particles; this was determined by viral RNA extraction and subsequent RT-qPCR. Virus incorporation efficiency reached 50% and the obtained corrected vaccine titer was 5 × 10^4^ TCID_50_ per dose. Particulate delivery system has the advantage to present multiple copies of an antigen – inactivated virus in our case—and promote trapping and retention of the selected antigen in the local draining lymphoid tissue (46). Additionally, particles are caught by antigen presenting cells (APC) leading to enhanced antigen presentation and the release of multiple cytokines promoting the induction of the adaptive immune response.

### PRRSV Viremia

One step RT-qPCR: RNA was purified from serum or tissue samples using the NucleoSpin RNA 8 virus kit (Macherey-Nagel, Düren, Germany) according to the manufacturer's instructions. PRRSV viremia was evaluated using a one-step RT-qPCR commercial kit (Tetracore, Rockville, MD, USA) following manufacturer's recommandations. Data were analyzed as relative expression or absolute quantification based on supplied PRRSV RNA positive control with known concentration in copies/mL. In some cases, unspecific amplifications were observed and no value was presented for some samples.

Flow cytometry: After infection, AMs were washed and permeabilized using commercial kit (eBioscience, San Diego, CA). Cells were incubated 20 min with mouse fluorescein (FITC) conjugate IgG1 antibody SR30 directed against PRRSV N protein (dilution 1/100) (RTI, Brookings, SD, USA), washed and a variable number of cells analyzed using Facscalibur (BD Biosciences).

### Detection of PRRSV-Specific Antibodies

ELISAs were performed on sera and nasal swabs sampled at 14, 28, and 42 days post-immunization to detect PRRSV-specific antibodies using a commercial kit (IDEXX, Toronto, Canada) according to the manufacturer's recommendations. Nasal swabs were incubated 1 h at room temperature in a PBS buffer completed with 1 mM phenylmethylsulfonyl fluoride (PMSF) before freezing stock at −20°C. Antibody titers were expressed as sample/positive (S/P) ratios. SP = [(absorbance value of sample in PRRSV well) - (absorbance value of sample in normal host cell well)]/[(mean absorbance value of positive serum control in PRRSV well) - (mean absorbance value of positive serum control in normal host cell well)]. All values are corrected by the negative control background.

### Peripheral Blood Mononuclear Cell IFNγ ELISPOT Analysis

Peripheral blood mononuclear cells (PBMC) were isolated from blood samples collected at day 28 and 42 post-immunization using Ficoll (Sigma–Aldrich, Oakville, ON, Canada) gradients. One day prior to PBMC isolation, nitrocellulose unifilter 350 microtiter plates (VWR, Radnor, PA, USA) were coated with monoclonal anti-porcine IFNγ antibody (Endogen, Rockford, IL, USA) in coating buffer at a concentration of 5 μg/mL for 16 h at 4°C and ELISPOT were performed as previously described (47–49). Briefly, 10^6^ or 10^5^ cells were stimulated with 100 μL of control medium, inactivated PRRSV at a MOI of 0.1 or concavalin A (Sigma–Aldrich) at 200 μg/mL (positive control). Plates were then incubated for 16 h at 37°C, washed, and incubated with rabbit anti-porcine IFNγ (Endogen, Rockford, IL, USA) at a concentration of 2 μg/mL for 16 h at 4°C. The plates were then washed and incubated with biotinylated-goat anti-rabbit immunoglobulin G (IgG) (H+L) (DiAMED, San Francisco, CA, USA) at a dilution of 1:5,000 for 2 h at room temperature. The wells were washed five times and incubated with streptavidin alkaline solution (Jackson ImmunoResearch, West Grove, PA, USA) at a dilution of 1:5,000 for 1.5 h at room temperature. After eight washes with double-distilled water, 5-bromo-4-chloro-3-indolyl phosphate/nitroblue tetrazolium (Sigma-Aldrich)-insoluble alkaline substrate solution was added (100 μL/well), and the plates were incubated for 5 min. The plates were then washed again with double-distilled water and dried overnight at room temperature. Spots were counted manually under an inverted light microscope. The number of spots observed in wells was evaluated with an AID ELISpot reader (AID GmbH, Strassberg, Germany) with subtracted background. Data were reported as the number of IFNγ-secreting cells per 10^6^ cells. When the quality of the sample or the number of cells was too low no data was generated explaining the small numbers of values (pigs) in some groups.

### Statistical Analysis

Statistical analyses were performed using the StatsModels and SciPy (50) Python packages. Two-way ANOVA (statsmodels v0.9.0) was used to assess the effects of poly(I:C) and peptides PR39, IDR-1002, and LL37 and the interaction between them. Two-way ANOVA was followed by *post-hoc* analysis with Tukey's HSD (see full statistical analysis in Supplementary Files). To compare the number of IFNγ secreting cells between groups, Welch's *t*-test (*P* < 0.05) was used with the Bonferroni correction. The group labels obey the following properties. Groups with entirely different labels (i.e., no shared letters) differed significantly from one another, while groups with the same labels or overlap between letters did not. Where the data exhibited significant positive skew, a log transform was applied to normalize the data before applying statistical tests. Visualizations were generated with the Matplotlib and Seaborn Python packages (51).

## Results

### Selected Peptides Interact With Synthetic Nucleic Acid Poly(I:C)

**Figure 1:**
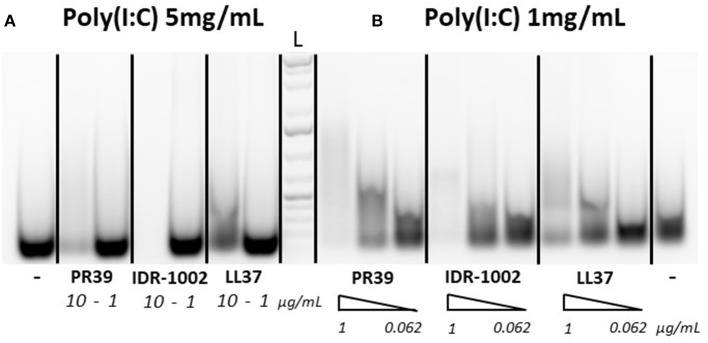
Peptide interactions with poly(I:C). Peptides were incubated with either 5 mg/mL **(A)** or 1 mg/mL **(B)** of poly(I:C) for 30 min at 37°C in 20 μL final volume and run on a 1% agarose gel. Poly(I:C) was detected with ethidium bromide. Peptides were used at concentrations of 10 or 1 μg/mL [**A** with 5 mg/mL poly(I:C)] or with a gradient (triangle shape symbol) of 1, 0.25, and 0.062 μg/mL [**B** with 1 mg/mL poly(I:C)]. A representative result of three experiments which provided similar results is shown. L, Ladder.

To assess whether the ability of selected peptides to inhibit PRRSV replication was due to modified cell signaling or direct interaction with poly(I:C), a gel migration analysis of poly(I:C)-HDP complexes was carried out to identify potential interactions between the peptides and poly(I:C). Since the interaction of LL37 with poly(I:C) was previously demonstrated by others (29), LL37 was used as a reference to assess and compare IDR-1002 and PR-39 peptides. Apparent reduced migration of poly(I:C) on agarose gel suggests interactions of the three peptides with poly(I:C) (Figures 1A,B). With high concentration of poly(I:C) (5 mg/mL), peptides PR39 and IDR-1002 seem to reduce more the migration of poly(I:C) than LL37 (Figure 1A). Similar observation was made when using lower concentration of poly(I:C) (Figure 1B).

### Some Peptides Can Reduce Poly(I:C)-Induced Response to Some Extent

**Figure 2:**
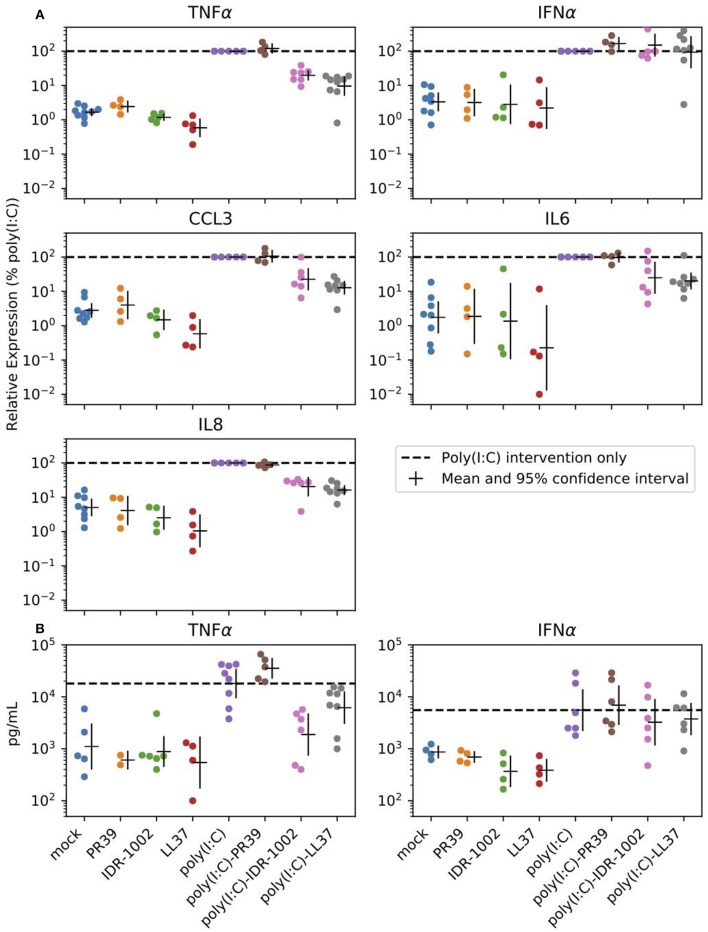
Peptide reduction of poly(I:C)-induced response. Selected peptides can reduce expression of poly(I:C) induced inflammatory cytokines in alveolar macrophages. Cells were stimulated with the positive control poly(I:C) (10 μg/mL), the HDPs (20 μg/mL) or a combination of both. **(A)** Transcript expression was analyzed 6 h after stimulation. **(B)** Cell supernatants were analyzed by ELISA to determine the production of TNFα and IFNα after 24 h of stimulation of the alveolar macrophages. Each group includes 4 to 8 pigs and estimated means with 95% confidence intervals are presented. The horizontal dashed line marks poly(I:C) intervention only (For statistical analysis please see Table 2).

The purpose of the next experiments was a brief assessment of some immunomodulatory properties of the peptides through their modulation of poly(I:C)-induced response in alveolar macrophages. These cells are antigen presenting cells and are the main targets and producers of the virus. When AMs were stimulated with poly(I:C) (10 μg/mL), high levels of TNFα, CCL3, IL6, and IL8 transcripts were measured after 6 h (Figure 2). CCL2, CCL5, IFNα, IFNβ, and SOCS1 transcripts were also expressed under poly(I:C) stimulation (Supplementary Figure 2 and Supplementary Table 1). The two-way ANOVA analysis showed significant differences between poly(I:C) and no-poly(I:C) groups for all measured cytokines (Table 2). Significant differences between peptide groups were found for all but IL6 and IFNα. After adjusting for the marginal effects of the two factors (i.e., poly(I:C) and peptides), a significant interaction between the factors remained for TNFα protein (pg/mL) (Table 2), suggesting that, with respect to TNFα, the immunomodulatory properties of poly(I:C) and peptides are not conferred by completely independent mechanisms, but through directly or indirectly interacting mechanisms of the immune activation pathway.

**Table 2:** Two-way ANOVA (type II sum of squares; statsmodels v0.9.0) was performed on the cytokine data.

	**sum_sq**	***df***	***F***	**PR(>F)**
**TNFalpha**				
C(Peptide)	3.858292	2	22.818448	**1.32E-06**
C(PolyIC	14.482661	1	171.304754	**1.87E-13**
C(Peptide):C(PolyIC)	0.340781	2	2.015422	1.52E-01
Residual	2.367211	28	NaN	NaN
**IFNalpha**				
C(Peptide)	0.269951	2	0.471966	6.29E-01
C(PolyIC)	20.118697	1	70.34873	**1.35E-08**
C(Peptide):C(PolyIC)	0.015425	2	0.026968	9.73E-01
Residual	6.863645	24	NaN	NaN
**CCL3**				
C(Peptide)	3.599368	2	14.92347	**6.15E-05**
C(PolyIC)	12.142635	1	100.690031	**4.60E-10**
C(Peptide):C(PolyIC)	0.066029	2	0.273766	7.63E-01
Residual	2.894261	24	NaN	NaN
**IL6**				
C(Peptide)	2.672236	2	2.389105	0.11318
C(PolyIC)	19.243249	1	34.408744	**0.000005**
C(Peptide):C(PolyIC)	0.605445	2	0.541297	0.588933
Residual	13.422111	24	NaN	NaN
**IL8**				
C(Peptide)	2.024658	2	9.246024	**1.05E-03**
C(PolyIC)	9.064626	1	82.791024	**3.01E-09**
C(Peptide):C(PolyIC)	0.208371	2	0.951568	4.00E-01
Residual	2.627713	24	NaN	NaN
**TNFalpha (pg/mL)**				
C(Peptide)	2.447746	2	6.925438	**0.004043**
C(PolyIC)	5.621733	1	31.811278	**0.000007**
C(Peptide):C(PolyIC)	2.120345	2	5.99912	**0.007447**
Residual	4.418034	25	NaN	NaN
**IFNalpha (pg/mL)**				
C(Peptide)	0.54701	2	1.670915	0.20922
C(PolyIC)	6.856436	1	41.887813	**0.000001**
C(Peptide):C(PolyIC)	0.003793	2	0.011587	0.988485
Residual	3.928457	24	NaN	NaN

*Bold values indicate cytokine transcripts and proteins (pg/mL)*.

### LL37 Reduces PRRSV Replication *in vitro*

**Figure 3:**
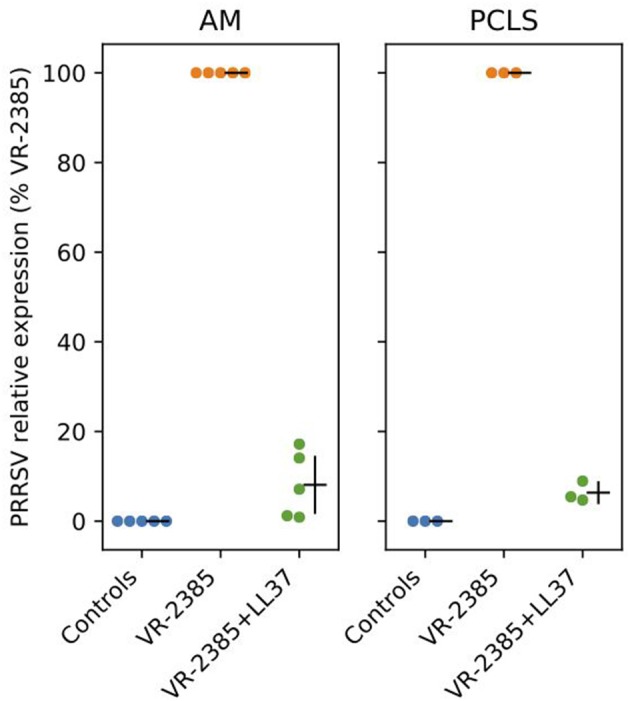
LL37 impacts on PRRSV replication *in vitro*. RT-qPCR was used to measure PRRSV VR-2385 replication after inoculation of the virus to AMs and PCLS *in vitro* with or without addition of LL37 (*n* = 3–5 pigs). AM, alveolar macrophage and PCLS, precision cut lung slices.

To determine whether selected peptides inhibit PRRSV replication, we infected AMs and lung tissues with PRRSV VR-2385 mixed 1 h in advance with IDR-1002, LL37, and PR39. As shown in a preliminary test (Supplementary Figure 3), an inhibition of PRRSV infection was observed in AMs when the virus was mixed with LL37 but not to a same extent with other peptides. Then, when LL37 was added to PRRSV VR-2385 on AMs and PCLS, a statistically significant decrease in viral replication was also observed by qPCR analyses (*P* < 0.05) (Figure 3).

### Assessment of LL37 and IDR-1002 Adjuvant Properties

HDPs have been used successfully as adjuvants previously in different species (1, 3, 9, 24–26). In our conditions, LL37 and IDR-1002 were able to significantly decreased TNFα protein production consecutive to an induction of innate immune response through poly(I:C) stimulation in AMs, the main target of PRRSV. That observation could be seen as disadvantage as well as an advantage in a perspective of using the peptides as adjuvants even if AMs are not the only antigen presenting cells in the host. Moreover, for several cytokines and transcripts relating to immune response we did not see any differences between poly(I:C) and poly(I:C) + peptides treatments complicating the interpretation of the data regarding the adjuvant potential of the peptides. Thus, because chemokine and cytokine expressions consecutive to poly(I:C) stimulation were mostly altered by LL37 and IDR-1002, we chose to test the adjuvant potential of these two peptides *in vivo* in association with inactivated PRRSV vaccine. An immunization trial against PRRSV was carried out and the adjuvant potential of LL37 and IDR-1002 was assessed after intra-muscular and intra-nasal immunizations. The two peptides were formulated with poly(I:C) and with 1 × 10^5^ TCID_50_ of inactivated PRRSV VR-2385 to produce microparticles using a simple assembly procedure previously described (26, 44). The microparticles ranged from 0.5 to 2 micron in size, facilitating the delivery of antigens to antigen-presenting cells as stated before.

**Figure 4:**
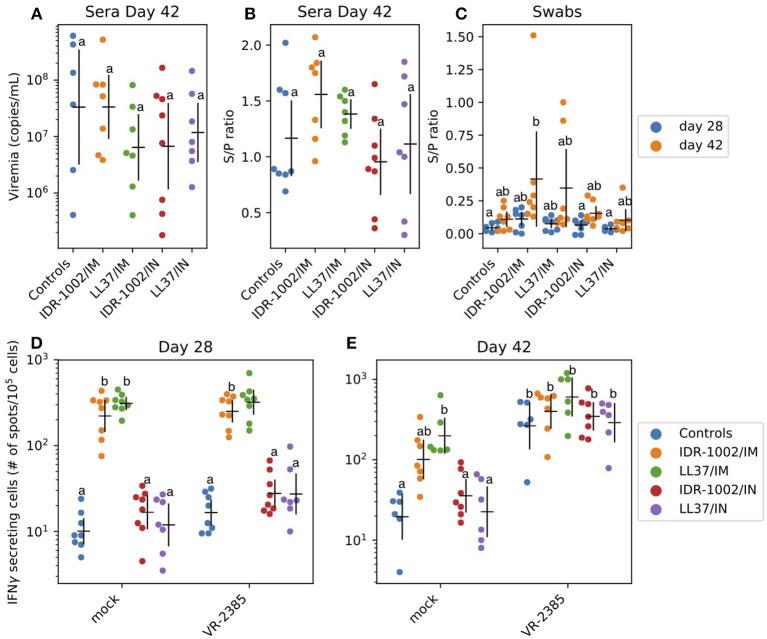
Adjuvant potential of LL37 and IDR-1002 in an immunization trial against PRRSV **(A–C)**. Serum virus titers and PRRSV specific antibody titers in serum as well as nasal swab presented as sample/positive ratio were determined (day 28 and/or 42) after challenge of the different vaccinated groups. For each group, including 5 to 8 pigs, the estimated mean with 95% confidence interval is presented. Labels (a/b) were derived using Tukey's HSD (*P* < 0.05). IFNγ production in PBMC 28 and 42 days after the first immunization **(D,E)**. IFNγ producing cells were analyzed before (day 28) and after (day 42) challenge. For each group, including 5–8 pigs, the estimated mean with 95% confidence interval is presented. Different letters (a/b) indicate significant differences (Welch's *t*-test; *P* < 0.05) between groups. In a comparison, if there is one letter in common—for instance two (a) or (ab) and (a), (b) and (ab) - it means there is no statically significant difference between the two conditions (*P* > 0.05). Conversely, if there is no letter in common in the comparison—for instance (a) and (b)—it means there is a statistically significant difference between the two conditions (*P* < 0.05).

After challenge, viremia was detected in all animals (Figure 4A, no statistically significant differences between groups – *P* > 0.05). All immunized animals developed moderate titers of PRRSV-specific antibodies with ratios between 0.5 and 2 (Figure 4B, no statistically significant differences between groups – *P* > 0.05). As expected, intra-muscular immunization resulted in higher serum antibody titers than intra-nasal immunization (Figure 4C: groups IDR-1002/IM and LL37/IM showing the highest S/P ratios). In nasal swab samples, antibody ratios were usually low (Figure 4C). However, an increase between day 28 and 42 was identified in all the groups. The immune responses consecutive to intra-muscular immunization were invariably better than after intra-nasal immunization regardless of the formulation used (Figures 4B,C).

IFNγ production in PBMC was analyzed at day 28 and 42 (Figures 4D,E). Non-specific stimulation of PBMC was identified at day 28 and 42 (200–500 spots/10^6^ cells vs. 10 in control group). At day 42, a specific anti-PRRSV response was identified. The group which received LL37 via the intra-muscular route (LL37/IM) showed a higher response to PRRSV VR-2385 than the other groups (Figures 4D,E). However, the difference was not statistically significant (*P* > 0.05) and non-purified PRRSV used as antigen in the ELISPOT assay might also have induced, to a low extent, non-specific IFNγ responses to stress antigens such as MHC class I polypeptide-related sequence A (MICA) expressed by virus-infected cells.

## Discussion

In the current study, we aimed at carrying out a first assessment of immunomodulatory and antiviral properties of LL37, PR39, and IDR-1002 in pigs. We identified some physical interactions of three peptides with poly(I:C) (Figure 1). Then, we assessed the alteration of AMs' poly(I:C)-induced response by the peptides. So far, broad transcriptomic analyses subsequent to stimulations with the peptides alone and focusing on interaction between HDPs and TLR ligands with limited analyses of inflammation markers have been performed by others (10, 11, 29, 52). With a focus on inflammatory cytokine transcripts associated with CCL3, IL6, IL8, and TNFα, we were able to confirm that LL37 and IDR-1002 can alter the cellular response to some extent (Figure 2 and Table 2). In addition, type I interferon, CCL2, and CCL5 transcripts were assessed and for all of them the upregulation induced by poly(I:C) was affected by neither HDP nor IDR-1002 treatments (Supplementary Figure 2). This observation was made at the transcriptional level. Also of note was the lack of PR39 interference with poly(I:C) stimulation in our conditions (Figure 2).

Because HDPs and their synthetic analog, IDR-1002, were able to interact with viral RNA analogs such as poly(I:C), we decided to evaluate the interaction of the peptides with a major porcine RNA virus. Using the PRRSV model, anti-PRRSV activity was identified for LL37. Viral replication was inhibited *in vitro* and *ex vivo* in PCLS (Supplementary Figure 3 and Figure 3). Interestingly, viral inhibition was not observed for IDR-1002 and PR39 despite IDR-1002 showing immunomodulatory properties after stimulation of AMs with poly(I:C). LL37 might have directly complexed with PRRSV nucleic acid inhibiting its replication in a direct fashion as already suggested (53). Alternatively, LL37, in a PRRSV context, might also have inhibited signaling pathways induced by the virus even if it seems less plausible based on a recent report (53) and our data. Indeed, we did not observe a clear impact of LL37, on its own, on mRNA expression in AMs. Thus, our data are in agreement with previous reports demonstrating antiviral properties of LL37 (21). In these studies, the authors associated antiviral properties to various mechanisms involving both viral particles and epithelial cells (21). More recently, another study also demonstrated antiviral properties for LL37 against human rhinovirus (53). In that report, authors showed that LL37 has antiviral activity when either the rhinovirus is exposed to the peptides before cell infection or after the cells have been infected (53). Moreover, they demonstrated that the action of cathelicidins, either from animal or human, is by directly affecting the virus rather than by an induction of apoptosis or necrotic cell death (53). Regarding the anti-PRRSV activity of some HDPs, only a few studies are available (35, 36). In these studies anti-PRRSV properties were demonstrated for pBD-3 (35), a porcine HDP of the β-defensin family, and PG-1 (36), a protegrin originally isolated from porcine leukocytes. To clarify the mechanism(s) controlling the anti-PRRSV properties of LL37 further studies are needed. The absence of viral inhibition of PR39 against PRRSV, in our conditions is puzzling since this porcine cathelicidin has also been associated with strong antiviral potential (21). Regarding IDR-1002, no direct antiviral activity has been described to our knowledge thus far, although indirectly IDR can increase the release of LL37 by neutrophils (54) with subsequent potential impact on virus particles.

Current vaccines against PRRS are still disappointing in terms of protection [for a review on PRRS see (32)]. In general, modified live attenuated vaccine (MLV) are more effective than their inactivated counterparts due to a better stimulation of the immune system (55, 56). However, they are still of concern due to possible spread and reversion to virulence through mutations of the vaccine virus. Regarding that point, inactivated vaccines are safer than MLV but, in general, less effective due to lower immunogenicity (56, 57). Thus, current research against PRRS is focused on alternative antiviral strategies (57) and on enhancing the efficacy of inactivated vaccines through the use of various adjuvants. With that idea in mind we assessed the potential of LL37 and IDR-1002 to serve as adjuvants in a vaccine formulation against PRRS. Indeed, based on our results *in vitro* and the literature presented above, both peptides could have an impact on the establishment of the adaptive immune response with their capacity to alter the innate cellular response, associated or not to concomitant antiviral properties. In our conditions, no protection was observed against the disease and only a few points can be reported (Figure 4). There were no clear differences between formulations including LL37 and IDR-1002. These observations were unexpected since interesting adjuvant properties were demonstrated for both peptides in different contexts (24–28, 58). As anticipated, using the IM route we observed a slightly better induction of the immune parameters we assessed than using the IN route. To explain these results some hypotheses can be formulated. First, the antigen dose could have been too low to induce a protective response in the host. Second, the presentation of the antigen could have been altered by the formulation resulting in a weak immune stimulation. The third possibility is that a cross-neutralization of peptides with TLR ligand agonists occurred, which has been previously reported (11, 29) and shown in this study. Fourth, correlates of protection for PRRSV are not always easy to assess, still need further investigations, and are strain dependent (32, 33). Finally, LL37 and IDR-1002 might not be suitable adjuvants on their own in the pig in a PRRSV vaccination context.

In conclusion, both antiviral and immunomodulatory properties could be identified for LL37, only immunomodulatory properties for IDR-1002, but both peptides failed to improve the immune response consecutive to an immunization with a killed vaccine in a PPRSV challenge experiment. Further studies are needed to decipher and explain these observed differences between the different HDP and their synthetic analogs.

## Data Availability

The raw data supporting the conclusions of this manuscript will be made available by the authors, without undue reservation, to any qualified researcher.

## Ethics Statement

Weaned Dutch Landrace pigs were purchased from Prairie Swine Centre, University of Saskatchewan. Animals were housed and cared in accordance with the guidelines of the Canadian Council for Animal Care. Experimental procedures are in accordance with the Animal Research Ethics Board (AREB) of the University of Saskatchewan (see also Materials and Methods section).

## Author Contributions

BL: designed and carried out experiments, analyzed data, and participated to the writing. DH: analyzed data, designed figures, performed statistical analysis, and participated to the writing. JvK and SS: carried out experiments. SW: carried animal experimentation. JZ: analyzed data and participated to the writing. FM: analyzed data, designed figures, and wrote the paper. VG: designed experiments, supervised BL, analyzed the data, and participated to the writing.

### Conflict of Interest Statement

The authors declare that the research was conducted in the absence of any commercial or financial relationships that could be construed as a potential conflict of interest.

## Supplementary Material

Supplementary Figure 1 Immunization trial—Body temperature was monitored daily for a total of 7 days until day 6 post-challenge in all the pig groups. IM, intra-muscular injection; IN, intra-nasal injection. Change in temperature over time was investigated by fitting a linear regression model to the data. The plots show the linear models of the groups together with their 95% confidence intervals. The *P*-value calculated for the regression coefficient using the *F*-test is shown in the top left corner of each plot.

Supplementary Figure 2 Peptide reduction of poly(I:C)-induced response – CCL2, CCL5, IFNβ, SOCS1, and STAT6. Selected peptides can reduce expression of poly(I:C) induced inflammatory cytokines in alveolar macrophages. Cells were stimulated with the positive control poly(I:C) (10 μg/mL), the HDPs (20 μg/mL) or a combination of both. Transcript expression was analyzed 6 h after stimulation. Each group includes 4 to 8 pigs and estimated means with 95% confidence intervals are presented. The horizontal dashed line marks poly(I:C) intervention only (For statistical analysis please see Supplementary Table 1).

Supplementary Figure 3 Preliminary analysis of peptide inhibition of PRRSV infection in AMs. Alveolar macrophage PRRSV infection was analyzed by SR30-FITC staining and flow cytometry analysis after 24 h of infection. Shown in gray are the control conditions (non-infected cells) and in white (infected cells) the PRRSV infection conditions.

Supplementary Table 1 Two-way ANOVA (type II sum of squares; statsmodels v0.9.0) was performed on the cytokine data.
