# Supplementary figures and images for: Synthetic Cationic Peptide IDR-1002 and Human Cathelicidin LL37 Modulate the Cell Innate Response but Differentially Impact PRRSV Replication in vitro
Source: Front Vet Sci. 2019 Jul 12;6:233. doi: 10.3389/fvets.2019.00233 (PMC6640542; doi:10.3389/fvets.2019.00233)

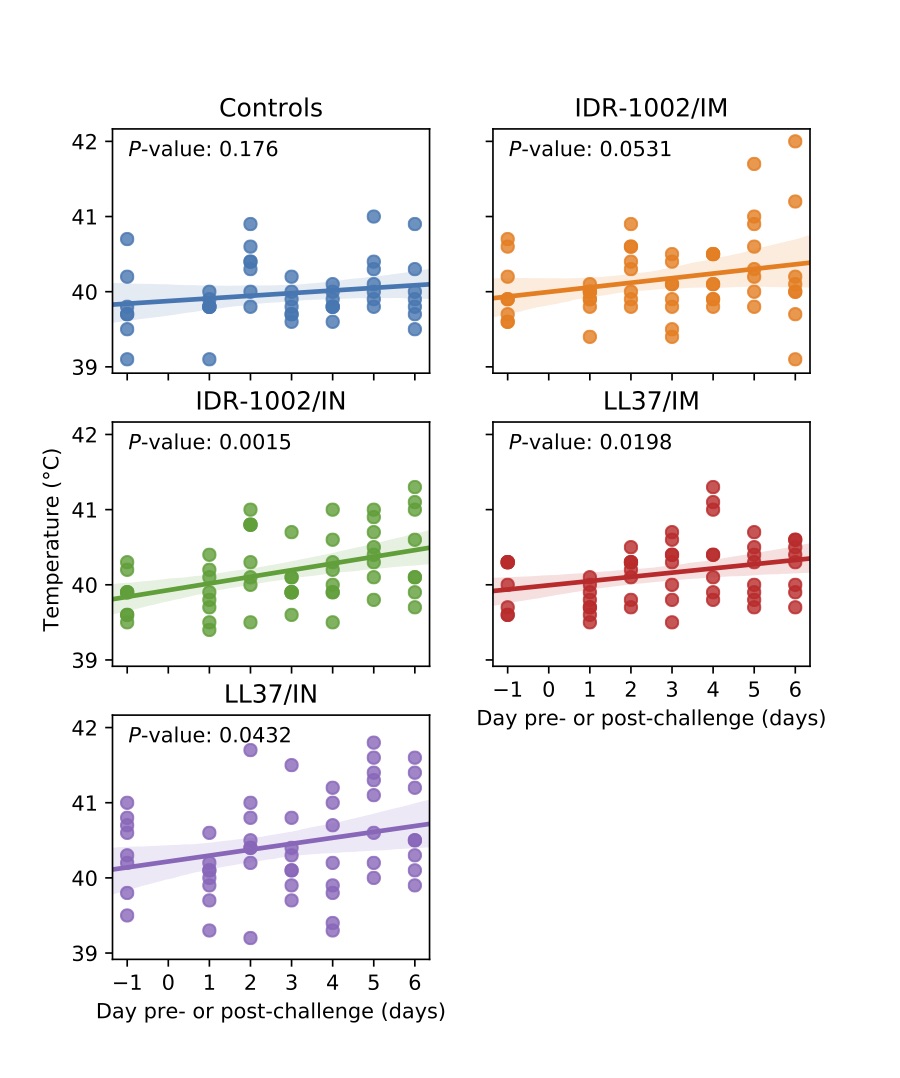

Supplement: Supplementary Figure 1 — Immunization trial—Body temperature was monitored daily for a total of 7 days until day 6 post-challenge in all the pig groups. IM, intra-muscular injection; IN, intra-nasal injection. Change in temperature over time was investigated by fitting a linear regression model to the data. The plots show the linear models of the groups together with their 95% confidence intervals. The P-value calculated for the regression coefficient using the F-test is shown in the top left corner of each plot. [file Image_1.JPEG]

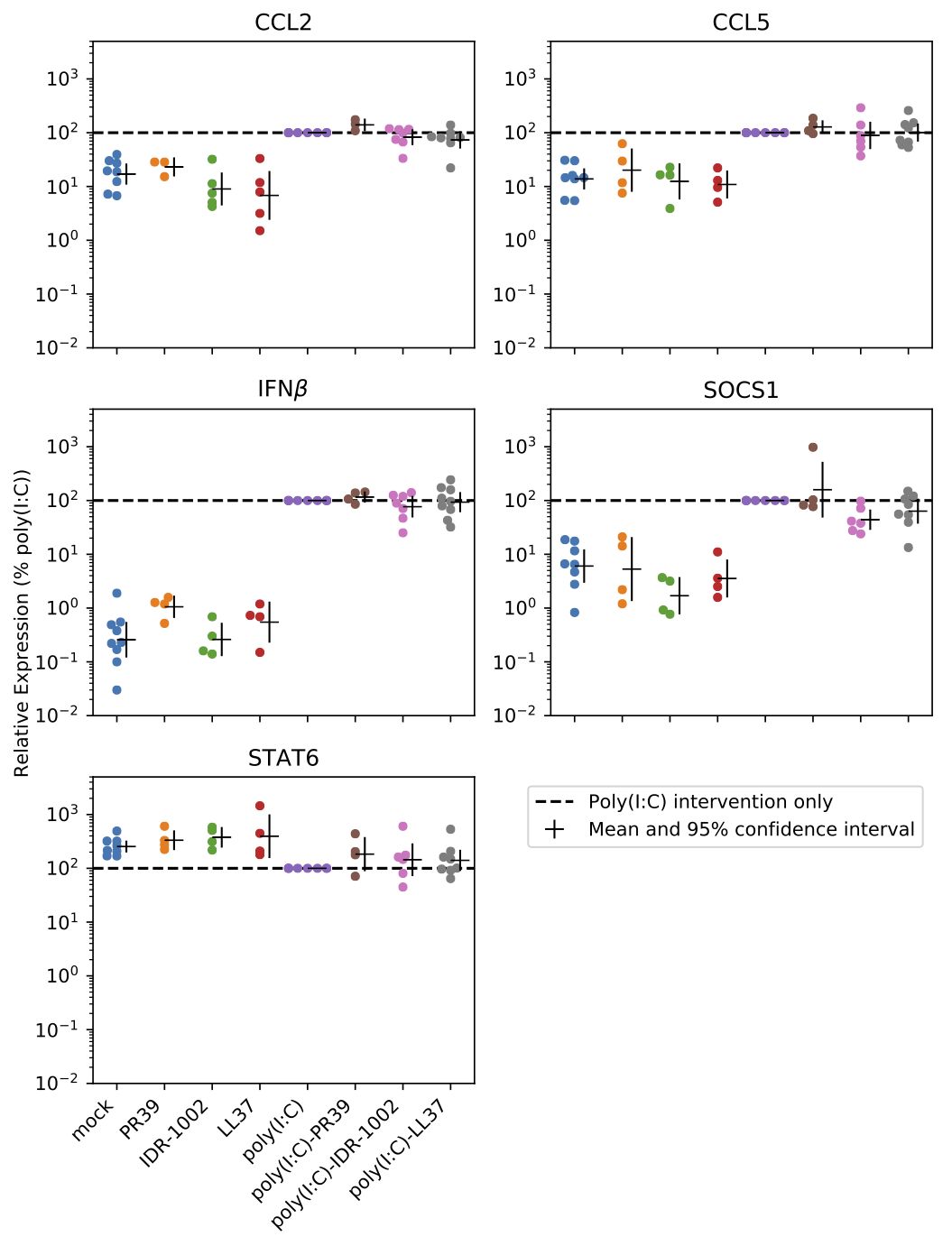

Supplement: Supplementary Figure 2 — Peptide reduction of poly(I:C)-induced response – CCL2, CCL5, IFNβ, SOCS1, and STAT6. Selected peptides can reduce expression of poly(I:C) induced inflammatory cytokines in alveolar macrophages. Cells were stimulated with the positive control poly(I:C) (10 μg/mL), the HDPs (20 μg/mL) or a combination of both. Transcript expression was analyzed 6 h after stimulation. Each group includes 4 to 8 pigs and estimated means with 95% confidence intervals are presented. The horizontal dashed line marks poly(I:C) intervention only (For statistical analysis please see Supplementary Table 1). [file Image_2.JPEG]

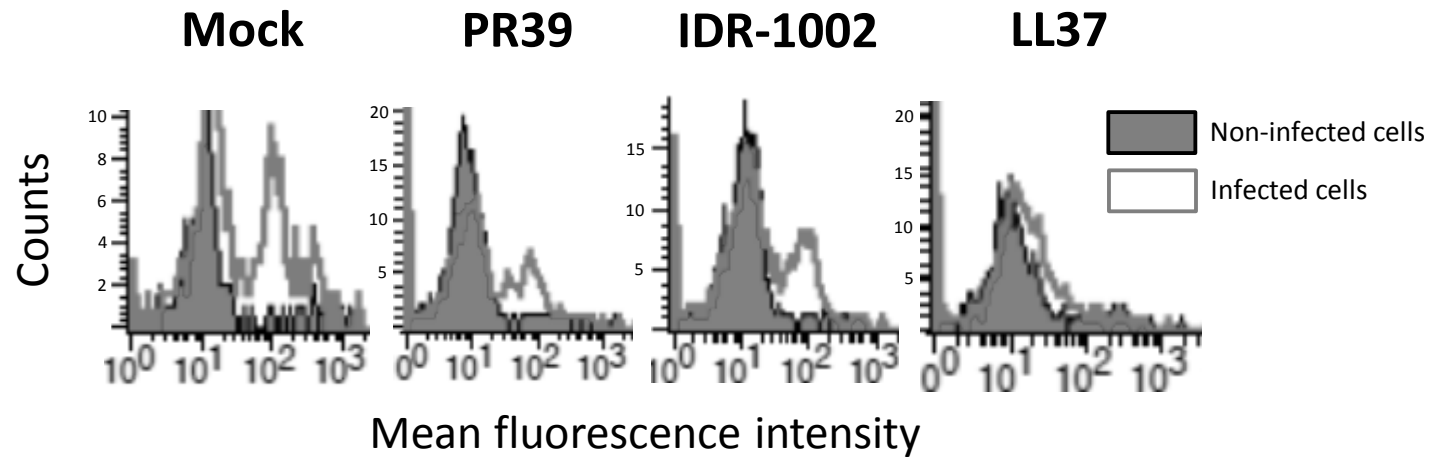

Supplement: Supplementary Figure 3 — Preliminary analysis of peptide inhibition of PRRSV infection in AMs. Alveolar macrophage PRRSV infection was analyzed by SR30-FITC staining and flow cytometry analysis after 24 h of infection. Shown in gray are the control conditions (non-infected cells) and in white (infected cells) the PRRSV infection conditions. [file Image_3.pdf]
