# Supplementary material for: Synthetic Cationic Peptide IDR-1002 and Human Cathelicidin LL37 Modulate the Cell Innate Response but Differentially Impact PRRSV Replication in vitro
Source: Front Vet Sci. 2019 Jul 12;6:233. doi: 10.3389/fvets.2019.00233 (PMC6640542; doi:10.3389/fvets.2019.00233)
Supplement: Supplementary file 5 [file Data_Sheet_1.PDF]

1 TNFα

1.1 Model Summary

```
                            OLS Regression Results
==============================================================================
Dep. Variable:              LogSignal   R-squared:                       0.879
Model:                            OLS   Adj. R-squared:                  0.858
Method:                 Least Squares   F-statistic:                     40.73
Date:                Thu, 06 Jun 2019   Prob (F-statistic):           5.32e-12
Time:                        10:31:11   Log-Likelihood:                -2.9449
No. Observations:                  34   AIC:                             17.89
Df Residuals:                      28   BIC:                             27.05
Df Model:                           5
Covariance Type:            nonrobust
==========================================================================================================
                                           coef    std err          t      P>|t|      [0.025      0.975]
----------------------------------------------------------------------------------------------------------
Intercept                                0.0728      0.130      0.560      0.580      -0.194       0.339
C(Peptide)[T.LL37]                      -0.3050      0.184     -1.659      0.108      -0.682       0.072
C(Peptide)[T.PR39]                       0.3143      0.195      1.611      0.118      -0.085       0.714
C(PolyIC)[T.True]                        1.2218      0.170      7.176      0.000       0.873       1.571
C(Peptide)[T.LL37]:C(PolyIC)[T.True]    -0.0078      0.235     -0.033      0.974      -0.490       0.474
C(Peptide)[T.PR39]:C(PolyIC)[T.True]     0.4712      0.267      1.765      0.088      -0.076       1.018
==============================================================================
Omnibus:                       29.702   Durbin-Watson:                   0.862
Prob(Omnibus):                  0.000   Jarque-Bera (JB):               70.071
Skew:                          -1.993   Prob(JB):                     6.09e-16
Kurtosis:                       8.794   Cond. No.                         10.7
==============================================================================

Warnings:
[1] Standard Errors assume that the covariance matrix of the errors is correctly specified.
```

1.2 ANOVA Summary

TNFα	sum_sq	df	F	PR(>F)
C(Peptide)	3.858292	2.0	22.818448	1.320918e-06
C(PolyIC)	14.482661	1.0	171.304754	1.870624e-13
C(Peptide):C(PolyIC)	0.340781	2.0	2.015422	1.521440e-01
Residual	2.367211	28.0	NaN	NaN

1.3 Post-Hoc Summary

```
      Multiple Comparison of Means - Tukey HSD,FWER=0.05
================================================================
      group1           group2      meandiff  lower  upper  reject
----------------------------------------------------------------
NoPolyIC-IDR-1002  NoPolyIC-LL37    -0.305   -0.867 0.2569 False
NoPolyIC-IDR-1002  NoPolyIC-PR39    0.3143  -0.2817 0.9104 False
NoPolyIC-IDR-1002 PolyIC-IDR-1002   1.2218   0.7015 1.7421  True
NoPolyIC-IDR-1002   PolyIC-LL37     0.9089   0.4133 1.4045  True
NoPolyIC-IDR-1002   PolyIC-PR39     2.0073   1.4113 2.6034  True
  NoPolyIC-LL37    NoPolyIC-PR39    0.6193   0.0233 1.2154  True
  NoPolyIC-LL37   PolyIC-IDR-1002   1.5268   1.0066 2.0471  True
  NoPolyIC-LL37     PolyIC-LL37     1.214    0.7184 1.7096  True
  NoPolyIC-LL37     PolyIC-PR39     2.3123   1.7163 2.9084  True
  NoPolyIC-PR39   PolyIC-IDR-1002   0.9075   0.3506 1.4644  True
```

```
  NoPolyIC-PR39      PolyIC-LL37    0.5946    0.0607 1.1286  True
  NoPolyIC-PR39      PolyIC-PR39    1.693     1.0647 2.3213  True
 PolyIC-IDR-1002     PolyIC-LL37   -0.3129   -0.7607 0.1349 False
 PolyIC-IDR-1002     PolyIC-PR39    0.7855    0.2286 1.3424  True
   PolyIC-LL37       PolyIC-PR39    1.0984    0.5644 1.6323  True
-----------------------------------------------------------------
```

2 IFNα

2.1 Model Summary

```
                            OLS Regression Results
==============================================================================
Dep. Variable:              LogSignal   R-squared:                       0.746
Model:                            OLS   Adj. R-squared:                  0.694
Method:                 Least Squares   F-statistic:                     14.13
Date:                Thu, 06 Jun 2019   Prob (F-statistic):           1.72e-06
Time:                        10:31:11   Log-Likelihood:                -20.444
No. Observations:                  30   AIC:                             52.89
Df Residuals:                      24   BIC:                             61.29
Df Model:                           5
Covariance Type:            nonrobust
=========================================================================================================
                                          coef    std err          t      P>|t|      [0.025      0.975]
---------------------------------------------------------------------------------------------------------
Intercept                               0.4512      0.267      1.687      0.105      -0.101       1.003
C(Peptide)[T.LL37]                     -0.1086      0.378     -0.287      0.776      -0.889       0.672
C(Peptide)[T.PR39]                      0.0517      0.378      0.137      0.892      -0.729       0.832
C(PolyIC)[T.True]                       1.7259      0.345      5.000      0.000       1.013       2.438
C(Peptide)[T.LL37]:C(PolyIC)[T.True]   -0.0986      0.476     -0.207      0.838      -1.081       0.883
C(Peptide)[T.PR39]:C(PolyIC)[T.True]   -0.0050      0.512     -0.010      0.992      -1.062       1.052
==============================================================================
Omnibus:                        6.245   Durbin-Watson:                   1.785
Prob(Omnibus):                  0.044   Jarque-Bera (JB):                4.875
Skew:                          -0.639   Prob(JB):                       0.0874
Kurtosis:                       4.505   Cond. No.                         11.1
==============================================================================

Warnings:
[1] Standard Errors assume that the covariance matrix of the errors is correctly specified.
```

2.2 ANOVA Summary

IFNα	sum_sq	df	F	PR(>F)
C(Peptide)	0.269951	2.0	0.471966	6.294423e-01
C(PolyIC)	20.118697	1.0	70.348730	1.352750e-08
C(Peptide):C(PolyIC)	0.015425	2.0	0.026968	9.734218e-01
Residual	6.863645	24.0	NaN	NaN

2.3 Post-Hoc Summary

```
       Multiple Comparison of Means - Tukey HSD,FWER=0.05
=================================================================
      group1           group2     meandiff  lower   upper  reject
-----------------------------------------------------------------
NoPolyIC-IDR-1002  NoPolyIC-LL37  -0.1086  -1.2778 1.0607 False
NoPolyIC-IDR-1002  NoPolyIC-PR39   0.0517  -1.1176 1.2209 False
NoPolyIC-IDR-1002 PolyIC-IDR-1002  1.7259   0.6586 2.7933  True
```

```
NoPolyIC-IDR-1002   PolyIC-LL37    1.5188   0.5062 2.5314  True
NoPolyIC-IDR-1002   PolyIC-PR39    1.7726   0.6033 2.9418  True
  NoPolyIC-LL37    NoPolyIC-PR39   0.1602   -1.009 1.3295 False
  NoPolyIC-LL37   PolyIC-IDR-1002  1.8345   0.7671 2.9019  True
  NoPolyIC-LL37     PolyIC-LL37    1.6273   0.6147 2.6399  True
  NoPolyIC-LL37     PolyIC-PR39    1.8811   0.7119 3.0504  True
  NoPolyIC-PR39   PolyIC-IDR-1002  1.6743   0.6069 2.7416  True
  NoPolyIC-PR39     PolyIC-LL37    1.4671   0.4545 2.4797  True
  NoPolyIC-PR39     PolyIC-PR39    1.7209   0.5516 2.8901  True
 PolyIC-IDR-1002    PolyIC-LL37   -0.2072  -1.1002 0.6859 False
 PolyIC-IDR-1002    PolyIC-PR39    0.0466  -1.0207 1.114  False
   PolyIC-LL37      PolyIC-PR39    0.2538  -0.7588 1.2664 False
-----------------------------------------------------------------
```

3 CCL3

3.1 Model Summary

```
                            OLS Regression Results
==============================================================================
Dep. Variable:              LogSignal   R-squared:                       0.831
Model:                            OLS   Adj. R-squared:                  0.796
Method:                 Least Squares   F-statistic:                     23.67
Date:                Thu, 06 Jun 2019   Prob (F-statistic):           1.48e-08
Time:                        10:31:11   Log-Likelihood:                -7.4911
No. Observations:                  30   AIC:                             26.98
Df Residuals:                      24   BIC:                             35.39
Df Model:                           5
Covariance Type:            nonrobust
=========================================================================================================
                                            coef    std err          t      P>|t|      [0.025      0.975]
---------------------------------------------------------------------------------------------------------
Intercept                                 0.1701      0.174      0.979      0.337      -0.188       0.528
C(Peptide)[T.LL37]                       -0.4062      0.246     -1.654      0.111      -0.913       0.101
C(Peptide)[T.PR39]                        0.4314      0.246      1.757      0.092      -0.075       0.938
C(PolyIC)[T.True]                         1.1838      0.224      5.281      0.000       0.721       1.646
C(Peptide)[T.LL37]:C(PolyIC)[T.True]      0.1578      0.309      0.511      0.614      -0.480       0.795
C(Peptide)[T.PR39]:C(PolyIC)[T.True]      0.2383      0.332      0.717      0.480      -0.448       0.925
==============================================================================
Omnibus:                        0.131   Durbin-Watson:                   0.967
Prob(Omnibus):                  0.937   Jarque-Bera (JB):                0.309
Skew:                          -0.120   Prob(JB):                        0.857
Kurtosis:                       2.565   Cond. No.                         11.1
==============================================================================

Warnings:
[1] Standard Errors assume that the covariance matrix of the errors is correctly specified.
```

3.2 ANOVA Summary

CCL3	sum_sq	df	F	PR(>F)
C(Peptide)	3.599368	2.0	14.923470	6.146141e-05
C(PolyIC)	12.142635	1.0	100.690031	4.596080e-10
C(Peptide):C(PolyIC)	0.066029	2.0	0.273766	7.628530e-01
Residual	2.894261	24.0	NaN	NaN

3.3 Post-Hoc Summary

Multiple Comparison of Means - Tukey HSD,FWER=0.05

group1	group2	meandiff	lower	upper	reject
NoPolyIC-IDR-1002	NoPolyIC-LL37	-0.4062	-1.1655	0.3531	False
NoPolyIC-IDR-1002	NoPolyIC-PR39	0.4314	-0.3278	1.1907	False
NoPolyIC-IDR-1002	PolyIC-IDR-1002	1.1838	0.4907	1.877	True
NoPolyIC-IDR-1002	PolyIC-LL37	0.9354	0.2778	1.5929	True
NoPolyIC-IDR-1002	PolyIC-PR39	1.8536	1.0944	2.6129	True
NoPolyIC-LL37	NoPolyIC-PR39	0.8376	0.0784	1.5969	True
NoPolyIC-LL37	PolyIC-IDR-1002	1.5901	0.8969	2.2832	True
NoPolyIC-LL37	PolyIC-LL37	1.3416	0.6841	1.9992	True
NoPolyIC-LL37	PolyIC-PR39	2.2598	1.5006	3.0191	True
NoPolyIC-PR39	PolyIC-IDR-1002	0.7524	0.0593	1.4455	True
NoPolyIC-PR39	PolyIC-LL37	0.504	-0.1536	1.1615	False
NoPolyIC-PR39	PolyIC-PR39	1.4222	0.6629	2.1815	True
PolyIC-IDR-1002	PolyIC-LL37	-0.2485	-0.8284	0.3315	False
PolyIC-IDR-1002	PolyIC-PR39	0.6698	-0.0233	1.3629	False
PolyIC-LL37	PolyIC-PR39	0.9182	0.2607	1.5758	True

4 IL6

4.1 Model Summary

OLS Regression Results

Dep. Variable:	LogSignal	R-squared:	0.610
Model:	OLS	Adj. R-squared:	0.528
Method:	Least Squares	F-statistic:	7.493
Date:	Thu, 06 Jun 2019	Prob (F-statistic):	0.000233
Time:	10:31:11	Log-Likelihood:	-30.504
No. Observations:	30	AIC:	73.01
Df Residuals:	24	BIC:	81.41
Df Model:	5		
Covariance Type:	nonrobust		

	coef	std err	t	P>|t|	[0.025	0.975]
Intercept	0.1327	0.374	0.355	0.726	-0.639	0.904
C(Peptide)[T.LL37]	-0.7791	0.529	-1.473	0.154	-1.871	0.312
C(Peptide)[T.PR39]	0.1385	0.529	0.262	0.796	-0.953	1.230
C(PolyIC)[T.True]	1.2636	0.483	2.618	0.015	0.267	2.260
C(Peptide)[T.LL37]:C(PolyIC)[T.True]	0.6855	0.665	1.030	0.313	-0.688	2.059
C(Peptide)[T.PR39]:C(PolyIC)[T.True]	0.4518	0.716	0.631	0.534	-1.026	1.930

Omnibus:	3.128	Durbin-Watson:	1.008
Prob(Omnibus):	0.209	Jarque-Bera (JB):	1.744
Skew:	0.497	Prob(JB):	0.418
Kurtosis:	3.637	Cond. No.	11.1

Warnings:
[1] Standard Errors assume that the covariance matrix of the errors is correctly specified.

4.2 ANOVA Summary

IL6	sum_sq	df	F	PR(>F)
C(Peptide)	2.672236	2.0	2.389105	0.113180
C(PolyIC)	19.243249	1.0	34.408744	0.000005
C(Peptide):C(PolyIC)	0.605445	2.0	0.541297	0.588933
Residual	13.422111	24.0	NaN	NaN

4.3 Post-Hoc Summary

```
      Multiple Comparison of Means - Tukey HSD,FWER=0.05
================================================================
     group1          group2     meandiff  lower  upper  reject
----------------------------------------------------------------
NoPolyIC-IDR-1002  NoPolyIC-LL37  -0.7791  -2.4142 0.856  False
NoPolyIC-IDR-1002  NoPolyIC-PR39   0.1385  -1.4966 1.7736 False
NoPolyIC-IDR-1002 PolyIC-IDR-1002  1.2636   -0.229 2.7562 False
NoPolyIC-IDR-1002   PolyIC-LL37     1.17    -0.246 2.586  False
NoPolyIC-IDR-1002   PolyIC-PR39    1.854    0.2189 3.4891  True
  NoPolyIC-LL37    NoPolyIC-PR39   0.9177  -0.7174 2.5527 False
  NoPolyIC-LL37   PolyIC-IDR-1002  2.0428   0.5501 3.5354  True
  NoPolyIC-LL37     PolyIC-LL37    1.9491   0.5331 3.3651  True
  NoPolyIC-LL37     PolyIC-PR39    2.6331   0.9981 4.2682  True
  NoPolyIC-PR39   PolyIC-IDR-1002  1.1251  -0.3675 2.6177 False
  NoPolyIC-PR39     PolyIC-LL37    1.0315  -0.3846 2.4475 False
  NoPolyIC-PR39     PolyIC-PR39    1.7155   0.0804 3.3506  True
 PolyIC-IDR-1002    PolyIC-LL37   -0.0936  -1.3425 1.1552 False
 PolyIC-IDR-1002    PolyIC-PR39    0.5904  -0.9022 2.083  False
   PolyIC-LL37      PolyIC-PR39    0.684    -0.732  2.1   False
----------------------------------------------------------------
```

5 IL8

5.1 Model Summary

```
                            OLS Regression Results
==============================================================================
Dep. Variable:              LogSignal   R-squared:                       0.797
Model:                            OLS   Adj. R-squared:                  0.755
Method:                 Least Squares   F-statistic:                     18.86
Date:                Thu, 06 Jun 2019   Prob (F-statistic):           1.29e-07
Time:                        10:31:11   Log-Likelihood:                -6.0419
No. Observations:                  30   AIC:                             24.08
Df Residuals:                      24   BIC:                             32.49
Df Model:                           5
Covariance Type:            nonrobust
==========================================================================================================
                                          coef    std err          t      P>|t|      [0.025      0.975]
----------------------------------------------------------------------------------------------------------
Intercept                               0.4033      0.165      2.438      0.023       0.062       0.745
C(Peptide)[T.LL37]                     -0.3839      0.234     -1.641      0.114      -0.867       0.099
C(Peptide)[T.PR39]                      0.2087      0.234      0.892      0.381      -0.274       0.692
C(PolyIC)[T.True]                       0.9062      0.214      4.243      0.000       0.465       1.347
C(Peptide)[T.LL37]:C(PolyIC)[T.True]    0.2832      0.294      0.962      0.346      -0.324       0.891
C(Peptide)[T.PR39]:C(PolyIC)[T.True]    0.4223      0.317      1.333      0.195      -0.232       1.076
==============================================================================
Omnibus:                        2.844   Durbin-Watson:                   2.152
Prob(Omnibus):                  0.241   Jarque-Bera (JB):                2.058
```

```
Skew:                          -0.642   Prob(JB):                        0.357
Kurtosis:                       3.016   Cond. No.                         11.1
==============================================================================

Warnings:
[1] Standard Errors assume that the covariance matrix of the errors is correctly specified.
```

5.2 ANOVA Summary

IL8	sum_sq	df	F	PR(¿F)
C(Peptide)	2.024658	2.0	9.246024	1.054006e-03
C(PolyIC)	9.064626	1.0	82.791024	3.005947e-09
C(Peptide):C(PolyIC)	0.208371	2.0	0.951568	4.002274e-01
Residual	2.627713	24.0	NaN	NaN

5.3 Post-Hoc Summary

```
      Multiple Comparison of Means - Tukey HSD,FWER=0.05
================================================================
     group1           group2     meandiff  lower  upper  reject
----------------------------------------------------------------
NoPolyIC-IDR-1002  NoPolyIC-LL37  -0.3839  -1.1074 0.3395 False
NoPolyIC-IDR-1002  NoPolyIC-PR39   0.2087  -0.5147 0.9322 False
NoPolyIC-IDR-1002 PolyIC-IDR-1002  0.9062   0.2458 1.5666  True
NoPolyIC-IDR-1002   PolyIC-LL37    0.8055   0.1789 1.432   True
NoPolyIC-IDR-1002   PolyIC-PR39    1.5372   0.8138 2.2607  True
  NoPolyIC-LL37    NoPolyIC-PR39   0.5927  -0.1308 1.3162 False
  NoPolyIC-LL37   PolyIC-IDR-1002  1.2901   0.6297 1.9506  True
  NoPolyIC-LL37     PolyIC-LL37    1.1894   0.5629 1.8159  True
  NoPolyIC-LL37     PolyIC-PR39    1.9212   1.1977 2.6446  True
  NoPolyIC-PR39   PolyIC-IDR-1002  0.6974   0.037  1.3579  True
  NoPolyIC-PR39     PolyIC-LL37    0.5967  -0.0298 1.2232 False
  NoPolyIC-PR39     PolyIC-PR39    1.3285   0.605  2.0519  True
 PolyIC-IDR-1002    PolyIC-LL37   -0.1007  -0.6533 0.4518 False
 PolyIC-IDR-1002    PolyIC-PR39    0.631   -0.0294 1.2915 False
   PolyIC-LL37      PolyIC-PR39    0.7318   0.1052 1.3583  True
----------------------------------------------------------------
```

6 TNFα (pg/mL)

6.1 Model Summary

```
                            OLS Regression Results
==============================================================================
Dep. Variable:              LogSignal   R-squared:                       0.724
Model:                            OLS   Adj. R-squared:                  0.669
Method:                 Least Squares   F-statistic:                     13.13
Date:                Thu, 06 Jun 2019   Prob (F-statistic):           2.51e-06
Time:                        10:31:11   Log-Likelihood:                -13.789
No. Observations:                  31   AIC:                             39.58
Df Residuals:                      25   BIC:                             48.18
Df Model:                           5
Covariance Type:            nonrobust
=========================================================================================================
                                          coef    std err          t      P>|t|      [0.025      0.975]
---------------------------------------------------------------------------------------------------------
Intercept                               2.9469      0.172     17.171      0.000       2.593       3.300
C(Peptide)[T.LL37]                     -0.2110      0.271     -0.778      0.444      -0.770       0.348
```

```
C(Peptide)[T.PR39]                      -0.1643      0.343     -0.479      0.636      -0.871       0.543
C(PolyIC)[T.True]                        0.3267      0.243      1.346      0.190      -0.173       0.827
C(Peptide)[T.LL37]:C(PolyIC)[T.True]     0.7282      0.354      2.058      0.050      -0.000       1.457
C(Peptide)[T.PR39]:C(PolyIC)[T.True]     1.4391      0.427      3.368      0.002       0.559       2.319
==============================================================================
Omnibus:                        1.664   Durbin-Watson:                   0.861
Prob(Omnibus):                  0.435   Jarque-Bera (JB):                1.511
Skew:                          -0.497   Prob(JB):                        0.470
Kurtosis:                       2.572   Cond. No.                         10.6
==============================================================================

Warnings:
[1] Standard Errors assume that the covariance matrix of the errors is correctly specified.
```

6.2 ANOVA Summary

TNFα (pg/mL)	sum_sq	df	F	PR(>F)
C(Peptide)	2.447746	2.0	6.925438	0.004043
C(PolyIC)	5.621733	1.0	31.811278	0.000007
C(Peptide):C(PolyIC)	2.120345	2.0	5.999120	0.007447
Residual	4.418034	25.0	NaN	NaN

6.3 Post-Hoc Summary

```
       Multiple Comparison of Means - Tukey HSD,FWER=0.05
=================================================================
     group1           group2     meandiff  lower  upper  reject
-----------------------------------------------------------------
NoPolyIC-IDR-1002  NoPolyIC-LL37   -0.211  -1.0473 0.6253 False
NoPolyIC-IDR-1002  NoPolyIC-PR39  -0.1643  -1.2221 0.8936 False
NoPolyIC-IDR-1002 PolyIC-IDR-1002  0.3267  -0.4213 1.0747 False
NoPolyIC-IDR-1002   PolyIC-LL37    0.8439   0.1442 1.5436  True
NoPolyIC-IDR-1002   PolyIC-PR39    1.6015   0.817  2.386   True
  NoPolyIC-LL37    NoPolyIC-PR39   0.0467  -1.0753 1.1687 False
  NoPolyIC-LL37   PolyIC-IDR-1002  0.5377  -0.2986 1.3739 False
  NoPolyIC-LL37     PolyIC-LL37    1.0549   0.2615 1.8483  True
  NoPolyIC-LL37     PolyIC-PR39    1.8125   0.9434 2.6816  True
  NoPolyIC-PR39   PolyIC-IDR-1002  0.4909  -0.5669 1.5488 False
  NoPolyIC-PR39     PolyIC-LL37    1.0082  -0.0161 2.0324 False
  NoPolyIC-PR39     PolyIC-PR39    1.7658   0.6819 2.8498  True
 PolyIC-IDR-1002    PolyIC-LL37    0.5173  -0.1824 1.2169 False
 PolyIC-IDR-1002    PolyIC-PR39    1.2749   0.4904 2.0594  True
   PolyIC-LL37      PolyIC-PR39    0.7576   0.019  1.4962  True
-----------------------------------------------------------------
```

7 IFNα (pg/mL)

7.1 Model Summary

```
                            OLS Regression Results
==============================================================================
Dep. Variable:              LogSignal   R-squared:                       0.653
Model:                            OLS   Adj. R-squared:                  0.581
Method:                 Least Squares   F-statistic:                     9.051
Date:                Thu, 06 Jun 2019   Prob (F-statistic):           6.11e-05
Time:                        10:31:12   Log-Likelihood:                -12.074
No. Observations:                  30   AIC:                             36.15
Df Residuals:                      24   BIC:                             44.55
```

```
Df Model:                           5
Covariance Type:            nonrobust
==========================================================================================================
                                          coef    std err          t      P>|t|      [0.025      0.975]
----------------------------------------------------------------------------------------------------------
Intercept                               2.5670      0.202     12.690      0.000       2.149       2.984
C(Peptide)[T.LL37]                      0.0190      0.286      0.067      0.948      -0.571       0.609
C(Peptide)[T.PR39]                      0.2745      0.286      0.960      0.347      -0.316       0.865
C(PolyIC)[T.True]                       0.9439      0.261      3.614      0.001       0.405       1.483
C(Peptide)[T.LL37]:C(PolyIC)[T.True]    0.0430      0.369      0.116      0.908      -0.719       0.805
C(Peptide)[T.PR39]:C(PolyIC)[T.True]    0.0529      0.369      0.143      0.887      -0.709       0.815
==============================================================================
Omnibus:                        0.028   Durbin-Watson:                   0.637
Prob(Omnibus):                  0.986   Jarque-Bera (JB):                0.172
Skew:                          -0.064   Prob(JB):                        0.917
Kurtosis:                       2.651   Cond. No.                         10.9
==============================================================================

Warnings:
[1] Standard Errors assume that the covariance matrix of the errors is correctly specified.
```

7.2 ANOVA Summary

IFNα (pg/mL)	sum_sq	df	F	PR(>F)
C(Peptide)	0.547010	2.0	1.670915	0.209220
C(PolyIC)	6.856436	1.0	41.887813	0.000001
C(Peptide):C(PolyIC)	0.003793	2.0	0.011587	0.988485
Residual	3.928457	24.0	NaN	NaN

7.3 Post-Hoc Summary

```
       Multiple Comparison of Means - Tukey HSD,FWER=0.05
================================================================
      group1           group2     meandiff  lower  upper  reject
----------------------------------------------------------------
NoPolyIC-IDR-1002  NoPolyIC-LL37   0.019   -0.8656 0.9036 False
NoPolyIC-IDR-1002  NoPolyIC-PR39   0.2745  -0.6101 1.1591 False
NoPolyIC-IDR-1002 PolyIC-IDR-1002  0.9439   0.1364 1.7514  True
NoPolyIC-IDR-1002   PolyIC-LL37    1.0059   0.1984 1.8134  True
NoPolyIC-IDR-1002   PolyIC-PR39    1.2713   0.4637 2.0788  True
  NoPolyIC-LL37    NoPolyIC-PR39   0.2555  -0.6291 1.1401 False
  NoPolyIC-LL37   PolyIC-IDR-1002  0.9249   0.1173 1.7324  True
  NoPolyIC-LL37     PolyIC-LL37    0.9869   0.1794 1.7944  True
  NoPolyIC-LL37     PolyIC-PR39    1.2522   0.4447 2.0597  True
  NoPolyIC-PR39   PolyIC-IDR-1002  0.6694  -0.1381 1.4769 False
  NoPolyIC-PR39     PolyIC-LL37    0.7314  -0.0761 1.5389 False
  NoPolyIC-PR39     PolyIC-PR39    0.9968   0.1892 1.8043  True
 PolyIC-IDR-1002    PolyIC-LL37    0.062   -0.6602 0.7843 False
 PolyIC-IDR-1002    PolyIC-PR39    0.3274  -0.3949 1.0496 False
   PolyIC-LL37      PolyIC-PR39    0.2653  -0.4569 0.9876 False
----------------------------------------------------------------
```
