# Supplementary material for: Synthetic Cationic Peptide IDR-1002 and Human Cathelicidin LL37 Modulate the Cell Innate Response but Differentially Impact PRRSV Replication in vitro
Source: Front Vet Sci. 2019 Jul 12;6:233. doi: 10.3389/fvets.2019.00233 (PMC6640542; doi:10.3389/fvets.2019.00233)
Supplement: Supplementary file 6 [file Data_Sheet_2.PDF]

1 CCL2

1.1 Model Summary

OLS Regression Results

Dep. Variable:	LogSignal	R-squared:	0.764
Model:	OLS	Adj. R-squared:	0.716
Method:	Least Squares	F-statistic:	16.15
Date:	Thu, 06 Jun 2019	Prob (F-statistic):	3.93e-07
Time:	11:21:18	Log-Likelihood:	-3.2841
No. Observations:	31	AIC:	18.57
Df Residuals:	25	BIC:	27.17
Df Model:	5		
Covariance Type:	nonrobust		

	coef	std err	t	P>|t|	[0.025	0.975]
Intercept	0.9538	0.134	7.120	0.000	0.678	1.230
C(Peptide)[T.LL37]	-0.1198	0.189	-0.633	0.533	-0.510	0.270
C(Peptide)[T.PR39]	0.4105	0.219	1.876	0.072	-0.040	0.861
C(PolyIC)[T.True]	0.9628	0.175	5.489	0.000	0.602	1.324
C(Peptide)[T.LL37]:C(PolyIC)[T.True]	0.0689	0.245	0.282	0.781	-0.435	0.573
C(Peptide)[T.PR39]:C(PolyIC)[T.True]	-0.1812	0.301	-0.602	0.553	-0.801	0.439

Omnibus:	2.258	Durbin-Watson:	0.759
Prob(Omnibus):	0.323	Jarque-Bera (JB):	1.114
Skew:	-0.058	Prob(JB):	0.573
Kurtosis:	3.921	Cond. No.	10.4

Warnings:
[1] Standard Errors assume that the covariance matrix of the errors is correctly specified.

1.2 Post-Hoc Summary

Multiple Comparison of Means - Tukey HSD,FWER=0.05

group1	group2	meandiff	lower	upper	reject
NoPolyIC-IDR-1002	NoPolyIC-LL37	-0.1198	-0.7037	0.464	False
NoPolyIC-IDR-1002	NoPolyIC-PR39	0.4105	-0.2637	1.0847	False
NoPolyIC-IDR-1002	PolyIC-IDR-1002	0.9628	0.4222	1.5033	True
NoPolyIC-IDR-1002	PolyIC-LL37	0.9118	0.3855	1.4382	True
NoPolyIC-IDR-1002	PolyIC-PR39	1.1921	0.5179	1.8663	True
NoPolyIC-LL37	NoPolyIC-PR39	0.5303	-0.1439	1.2045	False
NoPolyIC-LL37	PolyIC-IDR-1002	1.0826	0.542	1.6232	True
NoPolyIC-LL37	PolyIC-LL37	1.0317	0.5054	1.558	True
NoPolyIC-LL37	PolyIC-PR39	1.3119	0.6377	1.9861	True
NoPolyIC-PR39	PolyIC-IDR-1002	0.5523	-0.0848	1.1894	False
NoPolyIC-PR39	PolyIC-LL37	0.5014	-0.1236	1.1264	False
NoPolyIC-PR39	PolyIC-PR39	0.7816	0.0278	1.5354	True
PolyIC-IDR-1002	PolyIC-LL37	-0.0509	-0.5287	0.4269	False
PolyIC-IDR-1002	PolyIC-PR39	0.2293	-0.4077	0.8664	False
PolyIC-LL37	PolyIC-PR39	0.2802	-0.3448	0.9053	False

2 CCL5

2.1 Model Summary

OLS Regression Results

Dep. Variable:	LogSignal	R-squared:	0.733
Model:	OLS	Adj. R-squared:	0.677
Method:	Least Squares	F-statistic:	13.18
Date:	Thu, 06 Jun 2019	Prob (F-statistic):	3.10e-06
Time:	11:21:18	Log-Likelihood:	-2.1867
No. Observations:	30	AIC:	16.37
Df Residuals:	24	BIC:	24.78
Df Model:	5		
Covariance Type:	nonrobust		

	coef	std err	t	P>|t|	[0.025	0.975]
Intercept	1.0944	0.145	7.522	0.000	0.794	1.395
C(Peptide)[T.LL37]	-0.0570	0.206	-0.277	0.784	-0.482	0.368
C(Peptide)[T.PR39]	0.2094	0.206	1.018	0.319	-0.215	0.634
C(PolyIC)[T.True]	0.8551	0.188	4.552	0.000	0.467	1.243
C(Peptide)[T.LL37]:C(PolyIC)[T.True]	0.1103	0.259	0.426	0.674	-0.424	0.645
C(Peptide)[T.PR39]:C(PolyIC)[T.True]	-0.0512	0.279	-0.184	0.856	-0.626	0.524

Omnibus:	0.481	Durbin-Watson:	1.546
Prob(Omnibus):	0.786	Jarque-Bera (JB):	0.589
Skew:	0.069	Prob(JB):	0.745
Kurtosis:	2.328	Cond. No.	11.1

Warnings:
[1] Standard Errors assume that the covariance matrix of the errors is correctly specified.

2.2 Post-Hoc Summary

Multiple Comparison of Means - Tukey HSD,FWER=0.05

group1	group2	meandiff	lower	upper	reject
NoPolyIC-IDR-1002	NoPolyIC-LL37	-0.057	-0.6932	0.5793	False
NoPolyIC-IDR-1002	NoPolyIC-PR39	0.2094	-0.4268	0.8457	False
NoPolyIC-IDR-1002	PolyIC-IDR-1002	0.8551	0.2743	1.4359	True
NoPolyIC-IDR-1002	PolyIC-LL37	0.9085	0.3575	1.4594	True
NoPolyIC-IDR-1002	PolyIC-PR39	1.0133	0.3771	1.6495	True
NoPolyIC-LL37	NoPolyIC-PR39	0.2664	-0.3698	0.9026	False
NoPolyIC-LL37	PolyIC-IDR-1002	0.912	0.3312	1.4928	True
NoPolyIC-LL37	PolyIC-LL37	0.9654	0.4144	1.5164	True
NoPolyIC-LL37	PolyIC-PR39	1.0702	0.434	1.7065	True
NoPolyIC-PR39	PolyIC-IDR-1002	0.6456	0.0648	1.2264	True
NoPolyIC-PR39	PolyIC-LL37	0.699	0.148	1.25	True
NoPolyIC-PR39	PolyIC-PR39	0.8038	0.1676	1.4401	True
PolyIC-IDR-1002	PolyIC-LL37	0.0534	-0.4325	0.5393	False
PolyIC-IDR-1002	PolyIC-PR39	0.1582	-0.4226	0.739	False
PolyIC-LL37	PolyIC-PR39	0.1048	-0.4462	0.6558	False

3 IFNβ

3.1 Model Summary

OLS Regression Results

Dep. Variable:	LogSignal	R-squared:	0.950
Model:	OLS	Adj. R-squared:	0.940
Method:	Least Squares	F-statistic:	98.56
Date:	Thu, 06 Jun 2019	Prob (F-statistic):	4.74e-16
Time:	11:21:18	Log-Likelihood:	-1.1702
No. Observations:	32	AIC:	14.34
Df Residuals:	26	BIC:	23.13
Df Model:	5		
Covariance Type:	nonrobust		

	coef	std err	t	P>|t|	[0.025	0.975]
Intercept	-0.5834	0.139	-4.191	0.000	-0.870	-0.297
C(Peptide)[T.LL37]	0.3219	0.197	1.635	0.114	-0.083	0.727
C(Peptide)[T.PR39]	0.6069	0.197	3.083	0.005	0.202	1.012
C(PolyIC)[T.True]	2.4681	0.175	14.142	0.000	2.109	2.827
C(Peptide)[T.LL37]:C(PolyIC)[T.True]	-0.2371	0.242	-0.981	0.336	-0.734	0.260
C(Peptide)[T.PR39]:C(PolyIC)[T.True]	-0.4272	0.263	-1.624	0.116	-0.968	0.114

Omnibus:	1.574	Durbin-Watson:	1.465
Prob(Omnibus):	0.455	Jarque-Bera (JB):	1.452
Skew:	-0.474	Prob(JB):	0.484
Kurtosis:	2.565	Cond. No.	11.4

Warnings:
[1] Standard Errors assume that the covariance matrix of the errors is correctly specified.

3.2 Post-Hoc Summary

Multiple Comparison of Means - Tukey HSD,FWER=0.05

group1	group2	meandiff	lower	upper	reject
NoPolyIC-IDR-1002	NoPolyIC-LL37	0.3219	-0.283	0.9268	False
NoPolyIC-IDR-1002	NoPolyIC-PR39	0.6069	0.002	1.2119	True
NoPolyIC-IDR-1002	PolyIC-IDR-1002	2.4681	1.9319	3.0043	True
NoPolyIC-IDR-1002	PolyIC-LL37	2.5529	2.0388	3.067	True
NoPolyIC-IDR-1002	PolyIC-PR39	2.6478	2.0429	3.2527	True
NoPolyIC-LL37	NoPolyIC-PR39	0.2851	-0.3199	0.89	False
NoPolyIC-LL37	PolyIC-IDR-1002	2.1462	1.61	2.6824	True
NoPolyIC-LL37	PolyIC-LL37	2.231	1.7169	2.7451	True
NoPolyIC-LL37	PolyIC-PR39	2.3259	1.721	2.9308	True
NoPolyIC-PR39	PolyIC-IDR-1002	1.8611	1.3249	2.3974	True
NoPolyIC-PR39	PolyIC-LL37	1.946	1.4319	2.4601	True
NoPolyIC-PR39	PolyIC-PR39	2.0409	1.4359	2.6458	True
PolyIC-IDR-1002	PolyIC-LL37	0.0848	-0.3463	0.516	False
PolyIC-IDR-1002	PolyIC-PR39	0.1797	-0.3565	0.7159	False
PolyIC-LL37	PolyIC-PR39	0.0949	-0.4192	0.609	False

4 SOCS1

4.1 Model Summary

OLS Regression Results

Dep. Variable:	LogSignal	R-squared:	0.789
Model:	OLS	Adj. R-squared:	0.745
Method:	Least Squares	F-statistic:	17.94
Date:	Thu, 06 Jun 2019	Prob (F-statistic):	2.05e-07
Time:	11:21:18	Log-Likelihood:	-11.498
No. Observations:	30	AIC:	35.00
Df Residuals:	24	BIC:	43.40
Df Model:	5		
Covariance Type:	nonrobust		

	coef	std err	t	P>|t|	[0.025	0.975]
Intercept	0.2293	0.198	1.155	0.259	-0.180	0.639
C(Peptide)[T.LL37]	0.3205	0.281	1.142	0.265	-0.259	0.900
C(Peptide)[T.PR39]	0.4956	0.281	1.766	0.090	-0.084	1.075
C(PolyIC)[T.True]	1.4139	0.256	5.519	0.000	0.885	1.943
C(Peptide)[T.LL37]:C(PolyIC)[T.True]	-0.1614	0.353	-0.457	0.652	-0.890	0.567
C(Peptide)[T.PR39]:C(PolyIC)[T.True]	0.0621	0.380	0.164	0.871	-0.722	0.846

Omnibus:	0.552	Durbin-Watson:	0.917
Prob(Omnibus):	0.759	Jarque-Bera (JB):	0.665
Skew:	0.198	Prob(JB):	0.717
Kurtosis:	2.388	Cond. No.	11.1

Warnings:
[1] Standard Errors assume that the covariance matrix of the errors is correctly specified.

4.2 Post-Hoc Summary

Multiple Comparison of Means - Tukey HSD,FWER=0.05

group1	group2	meandiff	lower	upper	reject
NoPolyIC-IDR-1002	NoPolyIC-LL37	0.3205	-0.5472	1.1883	False
NoPolyIC-IDR-1002	NoPolyIC-PR39	0.4956	-0.3721	1.3634	False
NoPolyIC-IDR-1002	PolyIC-IDR-1002	1.4139	0.6218	2.2061	True
NoPolyIC-IDR-1002	PolyIC-LL37	1.573	0.8215	2.3245	True
NoPolyIC-IDR-1002	PolyIC-PR39	1.9717	1.1039	2.8395	True
NoPolyIC-LL37	NoPolyIC-PR39	0.1751	-0.6927	1.0429	False
NoPolyIC-LL37	PolyIC-IDR-1002	1.0934	0.3012	1.8856	True
NoPolyIC-LL37	PolyIC-LL37	1.2525	0.501	2.004	True
NoPolyIC-LL37	PolyIC-PR39	1.6512	0.7834	2.5189	True
NoPolyIC-PR39	PolyIC-IDR-1002	0.9183	0.1261	1.7105	True
NoPolyIC-PR39	PolyIC-LL37	1.0774	0.3259	1.8289	True
NoPolyIC-PR39	PolyIC-PR39	1.4761	0.6083	2.3438	True
PolyIC-IDR-1002	PolyIC-LL37	0.1591	-0.5037	0.8219	False
PolyIC-IDR-1002	PolyIC-PR39	0.5578	-0.2344	1.3499	False
PolyIC-LL37	PolyIC-PR39	0.3987	-0.3529	1.1502	False

5 STAT6

5.1 Model Summary

OLS Regression Results

Dep. Variable:	LogSignal	R-squared:	0.326
Model:	OLS	Adj. R-squared:	0.186
Method:	Least Squares	F-statistic:	2.323
Date:	Thu, 06 Jun 2019	Prob (F-statistic):	0.0743
Time:	11:21:18	Log-Likelihood:	-4.2581
No. Observations:	30	AIC:	20.52
Df Residuals:	24	BIC:	28.92
Df Model:	5		
Covariance Type:	nonrobust		

	coef	std err	t	P>|t|	[0.025	0.975]
Intercept	2.5761	0.156	16.524	0.000	2.254	2.898
C(Peptide)[T.LL37]	0.0206	0.220	0.093	0.926	-0.434	0.476
C(Peptide)[T.PR39]	-0.0543	0.220	-0.246	0.808	-0.509	0.401
C(PolyIC)[T.True]	-0.4161	0.201	-2.068	0.050	-0.832	-0.001
C(Peptide)[T.LL37]:C(PolyIC)[T.True]	-0.0324	0.277	-0.117	0.908	-0.605	0.540
C(Peptide)[T.PR39]:C(PolyIC)[T.True]	0.1580	0.299	0.529	0.602	-0.458	0.774

Omnibus:	2.009	Durbin-Watson:	1.104
Prob(Omnibus):	0.366	Jarque-Bera (JB):	1.453
Skew:	0.538	Prob(JB):	0.484
Kurtosis:	2.922	Cond. No.	11.1

Warnings:
[1] Standard Errors assume that the covariance matrix of the errors is correctly specified.

5.2 Post-Hoc Summary

Multiple Comparison of Means - Tukey HSD,FWER=0.05

group1	group2	meandiff	lower	upper	reject
NoPolyIC-IDR-1002	NoPolyIC-LL37	0.0206	-0.6611	0.7023	False
NoPolyIC-IDR-1002	NoPolyIC-PR39	-0.0543	-0.736	0.6274	False
NoPolyIC-IDR-1002	PolyIC-IDR-1002	-0.4161	-1.0385	0.2062	False
NoPolyIC-IDR-1002	PolyIC-LL37	-0.428	-1.0183	0.1624	False
NoPolyIC-IDR-1002	PolyIC-PR39	-0.3125	-0.9942	0.3692	False
NoPolyIC-LL37	NoPolyIC-PR39	-0.0749	-0.7566	0.6068	False
NoPolyIC-LL37	PolyIC-IDR-1002	-0.4367	-1.059	0.1856	False
NoPolyIC-LL37	PolyIC-LL37	-0.4485	-1.0389	0.1419	False
NoPolyIC-LL37	PolyIC-PR39	-0.333	-1.0147	0.3487	False
NoPolyIC-PR39	PolyIC-IDR-1002	-0.3618	-0.9841	0.2605	False
NoPolyIC-PR39	PolyIC-LL37	-0.3736	-0.964	0.2167	False
NoPolyIC-PR39	PolyIC-PR39	-0.2582	-0.9399	0.4235	False
PolyIC-IDR-1002	PolyIC-LL37	-0.0118	-0.5325	0.5089	False
PolyIC-IDR-1002	PolyIC-PR39	0.1037	-0.5186	0.726	False
PolyIC-LL37	PolyIC-PR39	0.1155	-0.4749	0.7059	False
